# Impact of ultrasound processing on alternative protein systems: Protein extraction, nutritional effects and associated challenges

**DOI:** 10.1016/j.ultsonch.2022.106234

**Published:** 2022-11-21

**Authors:** Rahel Suchintita Das, Brijesh K. Tiwari, Farid Chemat, Marco Garcia-Vaquero

**Affiliations:** a Section of Food and Nutrition, School of Agriculture and Food Science, University College Dublin, Belfield, Dublin 4, Ireland; b TEAGASC, Food Research Centre, Ashtown, Dublin 15, Ireland; c GREEN Team Extraction, UMR408, INRA, Université D'Avignon et des Pays de Vaucluse, Avignon Cedex, France

**Keywords:** Innovative technologies, Protein extraction, Nutritional properties, Allergenicity, Degradation

## Abstract

Proteins from alternative sources including terrestrial and aquatic plants, microbes and insects are being increasingly explored to combat the dietary, environmental and ethical challenges linked primarily to conventional sources of protein, mainly meat and dairy proteins. Ultrasound (US) technologies have emerged as a clean, green and efficient methods for the extraction of proteins from alternative sources compared to conventional methods. However, the application of US can also lead to modifications of the proteins extracted from alternative sources, including changes in their nutritional quality (protein content, amino acid composition, protein digestibility, anti-nutritional factors) and allergenicity, as well as damage of the compounds associated with an increased degradation resulting from extreme US processing conditions. This work aims to summarise the main advances in US equipment currently available to date, including the main US parameters and their effects on the extraction of protein from alternative sources, as well as the studies available on the effects of US processing on the nutritional value, allergenicity and degradation damage of these alternative protein ingredients. The main research gaps identified in this work and future challenges associated to the widespread application of US and their scale-up to industry operations are also covered in detail.

## Highlights

• US principles and parameters affecting protein extraction from alternative sources.
• US modes of operation and new technological developments for protein extraction.
• US impacts on protein quality, digestibility, antinutritional factors, and allergens.
• US effects on chemical degradation and oxidative damage of proteins.
• Prospects and challenges of upscaling US technology for protein recovery applications.

## Graphical abstract

**Figure:**
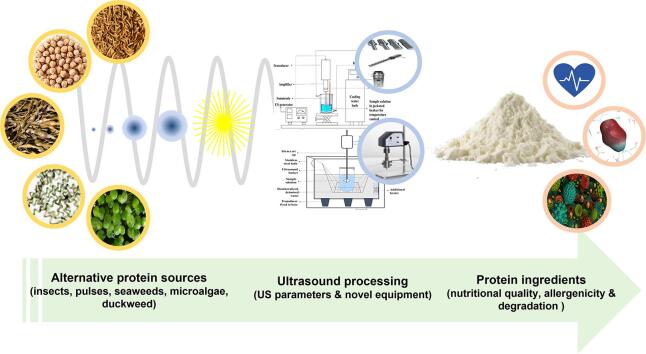

## 1 Introduction

Protein is a dietary macronutrient that fulfils an array of nutritional and physiological requirements of the human body [1]. Moreover, proteins are also credited for imparting a wide variety of organoleptic and physico-chemical characteristics when formulating new foods, via their functional properties [2]. Fabrication and commercialisation of protein enriched products for nutritional and functional applications are currently gaining considerable momentum in the food industry.

However, there is an unrelenting demand for sustainable and renewable food protein sources driven by the issues of escalating global population, increased urbanization, declining arable land availability, rising number of people affected by protein malnutrition, and stern environmental concerns [3], [4]. Demand for dairy, animal and fish proteins are on a constant rise with the global demand for animal proteins expected to double by 2050 [5]. However, animal rearing is associated with high greenhouse gas (GHG) emission [6], increased land and water usage, alongside recent concerns of increased risk of health issues related to red meat consumption along with ethical and religious disputes linked to animal slaughtering by a certain sector of the population [3], [7]. Hence, the shift has been embraced towards alternative protein that generally encompasses proteins derived from terrestrial plants (pulses, legumes, oilseeds, cereals, pseudo-cereals, nuts, fruit and vegetables, forage crops, etc.), aquatic sources (macroalgae, microalgae, duckweed), microbial sources (fungi, bacteria) and insects [8]. These alternative protein sources have several advantages compared to traditional sources, including reduced GHG emissions and carbon footprint during their production, low costs of production, high and efficient resource utilization (i.e. algae do not require arable land for cultivation and can be exploited through the year), and increased consumer acceptance and preference as the nutritional trends of veganism and/or vegetarianism and/or flexitarianism are increasing due to health and/or ethical concerns [3], [9], [10], [11], [12], [13]. Moreover, utilizing the waste streams and by-products arising from the processing of these alternative protein sources can add further value and contribute to the efficient utilisation of resources, reduction of the environmental burden of food production, ensure food security and promote the circularity and sustainability of the entire food system and the environment [3], [12], [14], [15].

These alternative protein sources are generally considered as nutritious, as they include a balanced proportion of essential and non-essential amino acids [8]. However, the presence of anti-nutritional factors (ANF) and toxic compounds [16], the comparatively lower protein quality when compared to animal proteins [7], and the resistance of some consumers towards non-conventional food sources (i.e. insects), along with associated regulatory challenges [17] are points that should be considered during their utilisation, processing and designing their future applications.

In this sense, the utilization of innovative technologies for extraction of protein and modification of their nutritional, structural and functional properties are being currently explored as a potential alternative to conventional techniques for the generation of protein ingredients from multiple sources. Innovative technologies help to combat the issues of long processing time, large volumes of chemical solvents, residual non-GRAS solvent concentration in extracts and higher cost and energy consumption associated with conventional chemical based extraction strategies [18], [19]. Ultrasound (US) has emerged as one of the most beneficial innovative techniques as it is considered to be safe, non-invasive, non-ionizing, effective, and ecologically-friendly option permitting clean label status of the treated product [19].

US technologies are based on the generation of mechanical waves with a frequency (>20 kHz), higher than the range audible to human hear (20 Hz to 20 kHz). These waves induce displacement of the particles of the medium through which they travel in a series of alternating compression (positive pressure) and rarefaction (negative pressure) [20]. Power US, characterized by high intensity (10 to 1000 W/cm^2^) and low frequency (20 to 100 kHz) waves, is responsible for inducing significant physical and chemical effects in food processing during extraction processes, commonly referred to as ultrasound-assisted extraction (UAE) [21]. High-intensity ultrasound (HIUS) is characterized by the phenomenon of acoustic cavitation [22]. At sufficiently high US intensity and power, the negative pressure of the rarefaction cycle may surpass the threshold inter-molecular force of attraction of the liquid medium to generate cavitation bubbles. Dissolved gases in the liquid will enter into the bubble during its expansion phase and will be retained to some extent even during the next compression phase. The bubbles will thereby grow by this process known as rectified diffusion [23]. Gradually, through multiple cycles, the bubbles will grow larger until they reach a critical size and become unable to withstand the internal vapour pressure. These bubbles will then violently rupture or collapse, releasing huge amount of energy which at the localised miniscule level produce instantaneous temperatures of about 5000 K and pressures of the order of 50 MPa [19]. There are 2 types of acoustic cavitation: (i) stable or non-inertial cavitation in which the bubbles are comparatively longer lived; and (ii) transient or inertial cavitation, in which the bubble hardly survive a cycle and collapse [19]. The physical effects of acoustic cavitation or sono-physical effects contributing to the UAE of proteins derive from the implosion of the cavitation bubbles generating turbulences, shock waves and strong shear forces along with simultaneous microstreaming characterised by strong eddies, microjets and vigorous non-linear bubble oscillations. These effects will induce fragmentation and de-texturization of the cellular matrix, surface erosion and sonoporation combined with sonocapillary effect that contribute formation of pores, deep micropits and channels in the cells [24]. All these will ultimately lead to hydration and swelling of the cells, cellular disruption and breakdown that will lead to increased surface area facilitating enhanced penetration of the extraction solvents and increased diffusional mass transfer of proteins into the extraction solvent [24]. This is the primary mechanism of how UAE helps in the process of protein extraction from various sources. The sono-chemical effects of US are related to the formation of highly reactive radicals, hydrogen (H•) and hydroxyl (OH•) radicals during the sonolysis of water in the medium, caused by the extreme conditions generated by acoustic cavitation. These radicals can initiate or participate in chemical reactions, such as oxidation of proteins [24]. Thus, both sono-physical and -chemical effects of US are responsible for extraction and for inducing changes in the structural, morphological, functional, thermal and nutritional properties of the protein achieved from myriad biomasses.

Several reviews have been published recently focusing on the role of US assisted extraction of proteins and the effects that US processing has on protein rich ingredients and formulated food products [25], [26], [27], [28], [29], [30], [31], [32], [33]. Most of these studies reported the effects of US on the functional properties of protein ingredients and protein rich foods of animal or plant origin [27], [28], [30], [31], [32], [33]; while others focused on reviewing US application and its influence on extraction, protein structure and functionality from plant sources [25], [26], [29]. However, advances in ultrasonics its synergistic effects with other technologies, mechanism and critical factors influencing extraction and processing strategies for protein from alternative sources including plant, algal, microbial and insect sources.

This review aims to put focus on the need of exploiting alternative protein resources (terrestrial, marine and air origin) and the potential of ultrasonication as a viable technology to cater to this purpose. The review discusses the fundamental US principles, physical parameters and design aspects of US devices including the combination of US with other technologies, as well as treatment medium and matrix parameters and how they impact extraction of proteins from alternative sources. The presence of ANF and allergens affect protein quality from alternative sources, the research being carried out to assay the role of US to reduce the ANF and allergens, as well as its role in improving digestibility and amino acid profile of the proteins are highlighted in this review. Contrastingly, undesirable chemical degradation processes reported in the literature, such as the oxidation damage of proteins which can be caused by power US, have also been discussed in this review. Lastly, the review draws attention to the prospects and challenges of upscaling US technology for protein recovery applications accompanied by strategies that could be possibly adopted to mitigate the associated hurdles.

## 2 US systems

US systems employed for processing are based on the delivery of waves emitted to the sample, these include direct and indirect contacts.

**Fig. 1:**
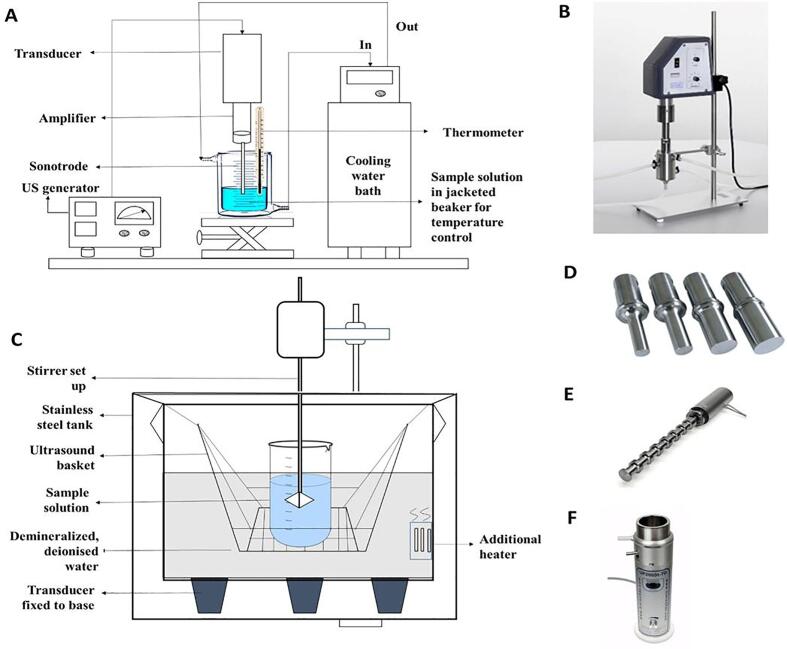
Image of different US systems that can be used for protein extraction. (A) US probe system for direct extraction; (B) Flow cell with probe for continuous extraction reprinted from Hielscher © 2022 www.hielscher.com; (C) US bath for indirect extraction (D) CupHorn from Hielscher © 2022 www.hielscher.com; (E) block sonotrodes from Hielscher © 2022 www.hielscher.com; and (F) Cascatrodes™ from Hielscher © 2022 www.hielscher.com.

Direct contact US refers to the fact that the sample solution is in direct contact with the US wave emitting device. This includes batch mode systems consisting of a probe/horn or sonotrode connected to a generator (see Fig. 1A) that produces alternating current of a specific frequency, followed by a transducer that transforms the electric current to mechanical displacements, and finally an amplifier which transfers the vibrations as a sound wave to the sonotrode [34]. Also, there are continuous mode systems consisting of an ultrasonic flow-through reactor chamber or flow cell in a closed system (see Fig. 1B) fitted to a sonotrode that allows both single pass and recirculation [35]. Flow cells enabling closed system for inline process, ensures safety and high processing uniformity and capacity as all material is continuously fed into the cavitational zone of the reactor chamber (see Fig. 1B). Flow-cells can be equipped with cooling jacket and heat exchanger to maintain the desired temperature and can be used to process high viscous liquids (up to 250.000 cP) (https://www.hielscher.com/ultrasonic-sonotrodes-flow-cells-accessories.htm, accessed on 11 June 2022).

Indirect contact US mostly refers to US baths in a batch set up containing sample solutions in containers placed in the liquid medium inside the bath. US waves are produced by the transducers, either attached to the bottom of the US bath or submerged inside the bath (see Fig. 1C). Once they are produced, US waves first pass through the liquid medium in the bath and then through the sample container’s wall to finally arrive inside the sample solution [34]. Supplementary heating can be carried out if the bath is fitted with additional heaters. Stirring of the sample solution can be provisioned through additional stirrer set up with the US device as seen in Fig. 1C.

Factors such as the probe type (e.g. stepped, linear taper and exponential taper shape), diameter of the probe, the immersion depth of the probe in the sample solution, and the positioning of the sample solution container inside the bath, play an important role in controlling the extraction of the target compounds including proteins, depending on the US systems used [24], [36]. For larger volumes, larger diameter probes are required, but with this increase in surface area achieved with larger probes, the ultrasonic intensity is lowered as the ultrasonic energy is transmitted over a larger area at a lower amplitude. However, the probes deliver quite high US intensity through the smaller surface of the probe’s tip compared to an US bath, as the latter having bigger surface area generates waves of much lower US intensity. Thus, the use of US probes generally lead to greater extraction efficiency, but it is accompanied by a rapid rise in the temperature of the treated solution that should be controlled by using a cooling jacket or ice bath [37]. When using probes, there is a decrease in US intensity along the radial and axial directions [24]. Thus, probe tips if not properly submerged at the middle depth of the liquid at the centre of the sample vessel, can incorporate air into the solution leading to foaming, impaired transmission of acoustic energy and reduced cavitation (https://www.sonics.com/vibracell/resources/frequently-asked-questions/ accessed on 11 June 2022). US baths on the other hand have the advantage of allowing the treatment of a large number of samples simultaneously unlike probe systems [24]. For the application of US at industrial scale, as mentioned in their website (https://www.hielscher.com/ultrasonic-sonotrodes-flow-cells-accessories.htm, accessed on 11 June 2022), Hielscher (Germany) has developed devices with 0.5–16 kW power equipped with block sonotrodes (see Fig. 1D) having a single horizontal surface area and Cascatrodes™ (see Fig. 1E) consisting of ring sonotrodes encasing the probe to provide an enlarged horizontal surface area. Another, US device designed by Hielscher (Germany) that can be used in both direct and indirect set up is the CupHorn (see Fig. 1F). This system can function as a sonotrode transmitting US waves directly into the sample; while on the other hand, it can also be filled with water to form a bath and containers with the sample solution can be placed in it for indirect sonication (https://www.hielscher.com/ultrasonic-sonotrodes-flow-cells-accessories.htm, accessed on 11 June 2022). Further modifications and advances in US equipment design including the use of booster amplifiers and different reactor geometries, such as a 4-finger sonotrode with 4 ultrasonic probe tips for simultaneous processing of 4 samples by Hielscher (Germany) (https://www.hielscher.com/ultrasonic-sonotrodes-flow-cells-accessories.htm, accessed on 11 June 2022) and equipment with automated frequency scanning [38], can further progress the scope of research and application of US assisted extraction of proteins.

Protein extraction from alternative sources has been commonly reported using temperature-controlled probe and bath systems. Flow cell coupled with a sonotrode has been reported for pilot-scale US-assisted protein extraction from soy okara [39]. A pilot-scale study of soy protein extraction using batch ultrasonic bath and continuous flow cell with inline sonotrode by MOULTON and WANG [40] revealed that continuous method extracted 54 % and 23 % more protein in case of water and alkali extraction respectively, over the batch method, both run under comparable processing volumes and duration. Also, to extract the same protein quantity, continuous process consumed 70 % less energy and sonication efficiency improved even with higher load of more viscous soy slurry [38], [40]. However, studies simultaneously comparing the efficiency of both bath and probe systems from alternative sources has not been extensively explored yet in protein extraction and processing. A recent study compared the modification of physicochemical and functional properties of de-phenolized sunflower meal protein and concluded that US probe (20 kHz) were more efficient compared to US bath (40 kHz) when extracting protein from this matrix [41].

Many authors have reported the diameter of the probe used for alternative protein extraction [42], [43], [44] and a handful have mentioned the probe immersion depth [44], [45]. But hardly any study reported the effects of different US probe diameters, variable probe immersion depths or different placement of the samples in a US bath, on the protein extraction efficiency of alternative proteins. Geometry and configuration of probes have direct implication on the distribution of acoustic energy, hence influence the extraction efficacy (discussed below). It should be noted that US may be attenuated by the geometry of the extractor/reactor in which the samples are placed, hence optimal reactor design is vital for efficient US delivery [46].

## 3 Effects of UAE conditions when applied to alternative protein sources

UAE can be used for the extraction of proteins from varied biomasses, either by using US as a pre-treatment or simultaneously during solid liquid extraction. The extraction yield and purity of protein obtained from the alternative protein sources during UAE or US pre-treatment prior to the main protein isolation process, are influenced by (1) the physical parameters of US and (2) the medium and matrix parameters [24], [26]. These factors when optimised, should not only help to achieve the highest possible efficiency of the protein extraction process, but also aim to make the process energy efficient, cost intensive and sustainable [24], [47].

### 3.1 Physical parameters

The influence of the physical parameters is associated either with the US waves generated (US frequency, power, intensity, power density, duty cycle) or with the design of the US equipment and its mode of operation are all relevant factors influencing US processing and all must be considered when applying US.

### 3.2 US frequency

This parameter refers to the number of passing waves per second, measured in hertz (Hz). The most frequently used range of frequency for extraction by US falls within 20 kHz to 100 kHz, unlike diagnostic imaging frequencies that are in the megahertz range [48]. Lower frequency US operate at higher intensity and since cavitation bubble size is also inversely related to frequency [49], low frequency high intensity acoustic waves produce larger sized transient cavitation bubbles, although less in number [50]. These large bubbles implode violently producing strong shear forces, microjets and turbulences [50]. All these phenomena help to enhance cellular degradation, solvent penetration, diffusion rate of proteins and consequently higher extraction yields [26], [51]. When using US waves at megahertz range, the intensity is not strong enough to prolong the rarefaction phase, making the compression-rarefaction cycles too short to induce the formation and growth of the cavitation bubbles [48], [52].

The US assisted alkaline isoelectric precipitation of soy protein was carried out using varying ultrasonic frequencies (20, 28, 35, 40, and 50 kHz) in a custom-made S-type five-frequency US equipment at a power density of 120 W/L, for 25 min, dilution ratio of 1:20 g/mL, pH 11.0 at 45 °C. At 28 kHz, soy protein extraction rate was at its highest, achieving protein extraction yields of 73.35 g protein/100 g. These extraction rates decreased to 16.63 g/100 g at 50 kHz which in turn had insignificant difference with the yields achieved at 40 kHz. The authors attributed this decrease in rate of protein extraction to the reduction in absolute sound pressure at the higher US frequencies. The authors also stated that their S-type five-frequency US extraction equipment based on a multi-physical-field coupling simulation system could improve the uniformity of the acoustic field distribution through frequency variation, as analysed through an acoustic field simulation model [53].

US systems can also be operated at sweeping frequencies, in which the ultrasonic frequency typically deviates symmetrically up and down from the central frequency during operation, as a function of time [54]. Sweeping the ultrasonic frequency strongly enhances the cavitation effect compared to that of fixed frequency mode [55], [56]. Sweeping frequency US (SFU) at 60 ± 1 kHz for sweeping period of 100 ms at 240 W, increased soluble protein content by 14.27 % during SFU-assisted soybean soaking [57]. SFU-assisted alkaline extraction using sweeping frequency of 28 ± 1.5 kHz at initial temperature of 25 °C and pH value of 9, sweep period of 100 ms, duty cycle of 77 % for 90 min improved the walnut protein yield by 34.9 % and the walnut protein content by 9.8 % [58].

**Fig. 2:**
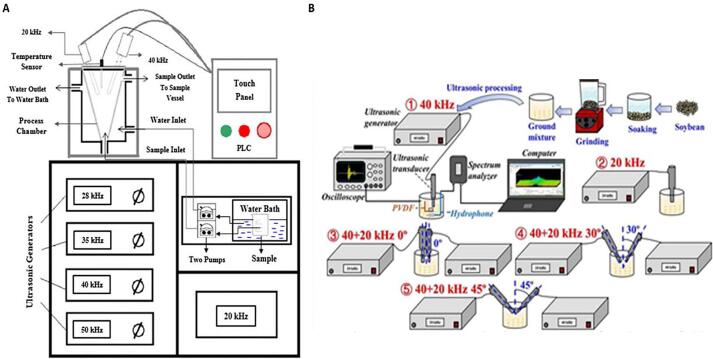
Representation of a (A) dual-frequency equipment adapted with permission from Dabbour, He [61]; and (B) Dual-frequency multi-angle equipment adapted with permission from Zhang, Wang [44].

Combining two or more frequencies in a single US system provides certain advantages due to the interaction between different US waves [59] as the combined influence of all the frequencies is higher than the effect of the sum of the individual frequencies [51], resulting in a significant increase in cavitation yield [60]. Pre-treatments using dual frequency of 20/40 kHz in a combined working mode and pulse on and off-time of 5 and 2 s respectively, was carried out in a specially designed equipment comprising of controllers, two probes, a temperature regulating system, a reaction vessel, and a liquid circulating system (Fig. 2A) followed by alkaline pH shift protein isolation on sunflower meal [61]. These conditions exhibited protein yield of 54.26 % with an overall energy consumption of 0.13 kW h at optimum US conditions of power density (220 W/L), temperature (45 °C) and extraction time (15 min) [61]. Soluble protein content in soy milk was analysed and compared between US treatments using US probes operating under multi-angle dual frequency US (40 + 20 kHz at angles of 0°, 30° and 45 deviated from the vertical baseline) with 2 m distance between the transducers, and single frequency probes operating at 40 and 20 kHz and placed vertically (0° away from the vertical baseline). Sweeping mode was used with sweeping amplitude of ± 1 kHz around the corresponding center frequency, and sweeping period of 100 ms [44] Fig. 2B). The tip diameter of the probe was 0.7–1.1 cm and it was placed at a depth of 1.5 cm inside the solution. Treatments at 40 + 20 kHz and 45° resulted in increases of soluble protein of 20.01 and 12.05 % compared to 40 kHz and 20 kHz treatments, respectively. Moreover, these treatments also achieved higher soluble protein contents compared to other treatments using the same frequency at other angles, with levels of 16.07 % higher soluble proteins compared to treatments at 40 + 20 kHz 0° and 19.02 % higher than those at 40 + 20 kHz 30° [44]. Multi frequency US models have also been effectively used for protein extraction from walnut meal [59] and the microalgae *Chlorella pyrenoidosa* [62].

### 3.3 US power

This parameter refers to the rate at which acoustic energy is emitted per unit time, expressed in watts or the total acoustic power percentage. It can be represented by the actual input power of the device dissipated as heat in the medium as shown in **Eq.** (1) measured by the calorimetric method assuming that the UAE system has no heat losses [19].

(1) $$P=m\cdot C_{P}\frac{\mathrm{dT}}{\mathrm{dt}}$$

Being *P*, the power in watt (W); *Cp*, the heat capacity of the solvent at constant pressure (J.g^−1^. °C^−1^); *m*, the mass of solvent (g); and *dT/dt*, the temperature rise per second.

An increase in protein yield, from approximately 0.25–0.3 mg protein/mL by conventional method (5–120 min) to levels ranging from approximately 0.25 to 0.4 mg/mL (5–40 min) from defatted rice bran was observed while increasing US power from 40, 60, 80 up to 100 W using a probe US with its horn tip immersed around 2 cm into the solution in a pulsed mode (pulse ON 59⁄OFF 10 s) and temperature controlled at 26.62 ± 2.74 °C in an US assisted alkaline protein extraction process [63]. Similar findings were reported when extracting albumin from rambutan seeds, with increases of protein extraction of 15 % when the US power increased from 0 to 20 W/g dry weight (DW) material, keeping sonication time constant (1 min) [64]. However, the authors did not appreciate any changes in protein extraction when increasing further the US power from 20 to 25 W/g DW [64]. Similarly, increase in US power from 100 to 400 W significantly enhanced the extraction yields of pecan protein to levels of up to 25.5 %, higher than those achieved initially (20.5 %); however, further increase to 500 W resulted in decreased extraction yield s below 25 % [65]. This decrease in protein extraction could be attributed to protein denaturation, hydrolysis or aggregation due to the formation of disulfide bridges through oxidation of sulphur containing amino acids in the protein induced by hydroxyl-free radicals generated at high US power [65].

### 3.4 US intensity

US intensity that is mainly considered for influencing the sono-chemical impact of US, is expressed as the energy transmitted per second or power transmitted per unit area of the emitting surface of the transducer as seen in **Eq.** (2). US intensity is directly correlated to the US power and amplitude of the transducer [66]. A minimum intensity is required to achieve the cavitation threshold; however, very high intensity and amplitude can lead to liquid agitation overtaking cavitation, impeding the wave propagation in the liquid medium [47].

(2) $$\mathrm{UI}=\frac{P}{S}$$

where, *UI* is the ultrasonic intensity (W/cm^2^), *P* is the US power (W, see **Eq.** (1)), and *S* is the area of the emitting surface of the transducer (cm^2^).

UAE using a probe (500 W maximum power, 15 mm probe immersion depth, 19 kHz frequency), at power levels (100, 300, and 500 W) and US intensity (75, 226, and 376 W/cm^2^) was applied for the extraction of soluble proteins from pineapple juice [67]. Higher protein contents were found in all sonicated samples compared to non-sonicated juice which can be attributed to protein release due to cell disruption caused by sonication. At US intensities > 226 W/cm^2^, the protein content decreased, reaching a minimum protein concentration of 57.88 ± 1.81 mg/L at the highest power intensity and the longest duration of US treatments (376 W/cm^2^ and 10 min) from the maximum protein concentration of 161.65 ± 1.12 obtained at 226 W/cm^2^ and 6 min, as the protein loss rate overcame the protein extraction rates [67].

### 3.5 US amplitude

US amplitude, when associated with the kinematic characteristics of the medium particles, indicates the maximum height of the wave cycle or the maximum amount of displacement of a particle of the medium from its resting position, thus, it is commonly expressed in µm [68], [69]. An increase in US amplitude is associated with an increase in the wave’s intensity and power, both being proportional to the square of the amplitude [69]. All these factors influence the energy associated with the collapse of the cavitational bubble that will be strongly enhanced at increased US amplitudes, also improving the extraction yields. High amplitudes can be useful when using viscous media, although these amplitudes can cause a rapid damage of the US transducers [70].

US amplitudes ranging from 25 to 35 % corresponding to vibration displacements of 28.5 μm and 39.9 μm were applied when extracting pea protein using an alkaline pH shift method assisted by US [71]. At US amplitude of 33.7 % and optimised pH shift parameters (pH 9.6, extraction time 13.5 min and dilution ratio of sample 1:11.5), the yields of protein extraction were at its highest (86.2 %), while the traditional method without the use of US achieved only 71.6 % [71].

### 3.6 US power density

Power density corresponds to the US power applied per unit volume of sample solution [72] (**Eq.** (3)). Power density is essential to control the cavitation effect as high power or high intensity when dissipated in a large volume of sample will not be effective in producing the cavitation effect as the US power density is decreased [73].

(3) $$\mathrm{PD}=\frac{P}{V}$$

Being *PD*, power density (W/cm^3^ or W/L); *P*, US power in watts (W); and *V*, volume of sample solution (cm^3^ or in L).

The influence of US power density during extraction of protein from soy flakes, soy flour, kidney bean and chickpea flour was studied by Byanju, Rahman [74]. The authors used an US probe (20 kHz, 750 W maximum power, 160 μm peak-to-peak amplitude) adjusting the US power density to 2.5 W/cm^3^ and 4.5 W/cm^3^ by varying the amplitude between 20 and 40 % and the volume of the samples. 2.5 and 4.5 W/cm^3^ power densities significantly increased protein extraction yields from soy flakes by 68.5 % and 90 %, respectively over the un-sonicated controls, with no significant increase for soy flours. In the case of kidney beans, protein extraction yield increased by 16.39 % at 4.5 W/cm^3^. On the contrary, chickpea, showed a reduction in the protein yield at the two US power densities, probably owing to protein-lipid interactions that increased the viscosity of the solution. The protein purity of all the samples decreased when using high power density due to co-extraction of other compounds, such as oil, phytoestrogens and sugars, along with protein due to intensified cavitation [74].

### 3.7 Duty cycle

**Fig. 3:**
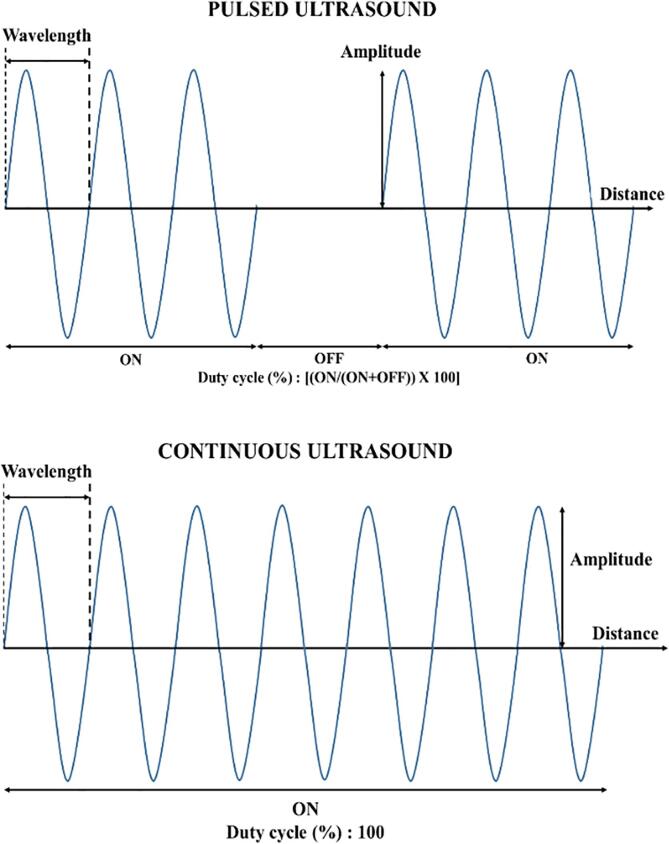
Illustration of the fundamental difference between pulsed and continuous US waveforms, with pulsed US waveform showing ON/OFF cycles and continuous US waveform as a continuous wave with no ON/OFF cycles.

When US is used in a pulsed mode, acoustic energy is transmitted in short bursts or pulses and average intensity of output over time is low; while in case of continuous US, acoustic energy is transmitted continuously, and acoustic intensity is constant over time [75]. In pulsed US, US output is cycled as ‘ON’ time which represents the pulse length and ‘OFF’ time is the pulse interval [76], as seen in Fig. 3. Duty cycle is the percentage of time during which the US signal is ‘ON’, and it is calculated by **Eq.** (4).

(4) $$\mathrm{DC}=\frac{\mathrm{ON}}{\mathrm{ON}+OFF}\times 100$$

For continuous US, duty cycle is 100 %. An increase in cavitation activity is observed during pulsed US over continuous US [77]. Also, it is believed that the residual cavitation nuclei, created after each pulse duration and persisting in the solution during the pulse interval, incites the formation of new cavitation bubbles during the next pulse ON time. The use of pulsed US at optimum pulse intervals can reduce energy consumption of US processes compared to continuous US [78].

Li, Yang [45] explored the extraction of protein from milled brewers spent grain using different duty cycles: 20 % (pulse ON 1/OFF 4 s), 40 % (pulse ON 2/OFF 3 s), 60 % (pulse ON 3/OFF 2 s), 80 % (pulse ON 4/OFF 1 s) and 100 % (pulse ON 5/OFF 0 s) at 250 W for 20 min. Although the protein extraction yields initially increased and peaked at 80 %, no significant increase was reported from a raise in duty cycle from 60 to 100 % and these extraction yields experiences a slight decrease at 100 % under continuous mode. Moreover, prolonged duty cycles could increase the thermal effect, leading to protein denaturation, aggregation and insolubilization, prompting the authors to choose 60 % duty cycle as the optimum processing condition [45].

Overall, it can be inferred from the reviewed studies, that in solid–liquid systems the sono-chemical and sono-physical effects of US improves extraction yields. However, after a certain threshold, protein extraction yields can either remain unchanged as the systems reach saturation or even decreases, if leaching of other components is induced by US, reducing the protein purity and creating undesirable protein aggregation.

### 3.8 Medium and matrix parameters affecting UAE

Medium parameters are those associated with the space through which ultrasonic waves are transmitted the matrix from the emitting source, while matrix parameters refer to the ones related to the raw material from which protein is being extracted.

#### 3.8.1 Temperature and time of extraction

There are variable results concerning the influence of temperature and US time on the extraction yields of protein from alternative sources. While an increase in medium temperature favours the increase of diffusion rate of target compounds in the solution, high temperatures also reduce the viscosity and surface tension of the solutions. High temperatures also help to disrupt the bonds that hold these compounds in their source matrix, thus enabling their faster release; but an undesirable climb in temperature increases solvent vapour pressure which causes more solvent vapour to fill in the cavitation bubbles which thereafter collapse with lesser intensity reducing the impact of cavitation [48], [79]. Moreover, high temperatures can also lead to protein denaturation and an increased cost of the processes due to energy consumption. During the UAE extraction of pecan protein, the protein extraction yields increased gradually with increases in the temperature, achieving the highest yield of protein extraction (27.05 ± 1.14 %) at 55 °C [65]. When the extraction temperature was further increased to 60 °C, the chemical bonds and molecular structure of proteins collapsed, resulting in a decrease in the protein yields. The authors also reported the effect of extraction time on the yields of protein extraction. While varying US duration from 5 to 15 min, protein extraction yield reached a maximum (25.59 ± 1.14 %) at 15 min and decreased with further increase in time of extraction [65]. Prolonged US treatments may lead to excessive cavitation and protein aggregation, resulting in a reduction of the mass transfer and decreased extraction yields of protein [80]. A similar pattern was observed during alkaline extraction of soluble proteins from wampee seeds assisted by US in a bath mode (240 W, 40 kHz) [81]. The authors achieved an initial increase in the extraction yield as the time of US increased, until reaching a maximum yield of extraction (15.06 %) at 64 min, while the yields decreased with further time of US extraction [81]. When exploring US pre-treatment times ranging from 1.5 to 3.5 min to achieve high yields of protein from tomato seed press cake, the optimum extraction time was found to be 2 min and 18 s using US at 210 W and 24 kHz [82]. Similarly, the highest protein yield from defatted rice bran using fixed US power at 15 W/g was noted at 2 min followed by a slight decrease in the protein yield when the time of extraction increased from 2 to 5 min [83]. On the contrary, soluble protein concentration of kiwifruit decreased continuously with increased sonication time (0, 4, 8, 12 and 16 min) when using US systems at 20 kHz, 400 W and 50 % duty cycle and exhibited approximately 20 % reduction from the untreated sample after 16 min. The decrease in soluble protein concentration was associated to the alteration of the secondary and tertiary protein structures induced by US, as well as to the formation of macromolecular protein aggregates [84]. Kingwascharapong, Chaijan [85] used an US pre-treatment to extract protein from defatted Bombay locust powder in water (1:20). The authors used a flat tip probe (maximum 70 µm at 100 % amplitude) at 750 W, 20 kHz, pulse ON 5 s/OFF 5 s at variable US amplitudes (40, 60 or 80 %) and times (10, 20 or 30 min). An increase in amplitude facilitated the increase in protein extraction yields at 30 min of extraction time and decreased at 10 min. These results could be attributed to the leaching or co-extraction of other components along with protein at high US amplitudes. Nevertheless, using the same US amplitude, >20 min of sonication did not significantly increase the protein recovery yields [85]. Overall, it has been observed that with increased sonication temperature and time, the protein extraction yield experiences an initial increase until reaching a plateau or it decreases with further increase in these extraction parameters.

#### 3.8.2 pH and type of solvent for extraction

The most commonly used solvents for extracting protein are alkaline solutions which leads to the solubilisation of the protein in solution from the solid matrix, followed by acid precipitation at the isoelectric point of the proteins. US is normally used during the protein solubilisation stage of the extraction process, after adjusting the pH to the alkaline value or applied as a pre-treatment of the sample slurry. With an increase in pH towards the alkaline range, disulfide, amide and hydrogen bonds are broken in the proteins leading to the release of the hydrogen ions from the carboxylic and sulphate groups, which contributes to the loosening of the molecules from the matrix and an enhancement in the rate of protein extraction [86], [87], [88]. Moreover, these modifications transform the surface charge of the proteins, making them negatively charged and improving their water solubility [89]. However, high pH may also be detrimental to the quality of proteins, as alkaline extraction can lead to protein denaturation [90], reduction in protein purity, darkening of colour [91] and formation of undesirable substances, such as lysinoalanine [92]. Few authors have reported the natural pH of around 6.8–7 to be the optimum pH for protein extraction from soy, peanut and the microalgae *Arthrospira platensis* [88], [93], [94]. An increase in the yield of protein extraction from *Dolichos lablab* L. with increased pH during US assisted alkaline extraction has been reported by Zhao, Wen [87]; however, several studies report an initial increase in protein yield with an increased pH, followed by a decrease at higher pH values [80], [91]. On the other hand, Nguyen and Le [88] also reported a significant decrease in the protein yield of defatted peanut meal by 7 % when increasing pH of the sample solution from 7 to 10.

Apart from using water and alkali as solvents for protein extraction, few studies have employed other solvents, such as Tris HCl buffer, ethanol, sodium phosphate buffer for UAE from moringa leaves [95], walnut meal [59] and *Arthrospira platensis* [96], respectively. Universally, the solvent should be judiciously selected so that, apart from being non-toxic, it has low vapour pressure, viscosity, and surface tension to facilitate high-intensity cavitation [46].

#### 3.8.3 Solid: Solvent ratio for extraction

Solid: solvent ratio or dilution ratio, when increased, usually helps to increase the extraction yields as the concentration gradient between the solvent and the extraction matrix contributes to increase the mass transfer of compounds [97], [98]. However, after a certain point, if saturation of the solvent occurs and there is an increase in viscosity of solution, there will be no further increase in the yields of extraction due to a reduction of the mass transfer [99].

Hadidi, Khaksar [80] analysed the influence of the solid: solvent ratio, ranging from 1:30 to 1:50 (w/v) when extracting protein from alfalfa in an US bath (20 kHz, 100 W). The authors achieved increased protein yields when increasing the solid: solvent ratio from 1:20 to 1:45, but the yields decreased with further increases in the solid: solvent ratios after reaching its peak [80]. Similar results on the influence of solid: solvent ratio were achieved when extracting protein from wampee seeds [81], quinoa [91] and pea [71]. Liu, Ma [81] reported that the extraction yield of wampee seed protein was increased initially and then it was reduced with the increase of solid:solvent ratio. The authors pointed out that although higher solid:solvent ratio facilitated diffusion, due to the increased concentration gradient between solid and solvent, it also was responsible for dampening the ultrasonic energy density per unit volume in the extraction solution. He, Wang [91] achieved maximum protein extraction yields (71.44 ± 1.49 %) using a solid:solvent ratio of 1:15, which decreased with the addition of further solvent. Wang, Zhang [71] achieved similar results when extracting pea protein by an alkaline isoelectric precipitation method supported by pulsed US (750 W pulse 5 s/5s). The authors while varying the solid:solvent ratio from 1:6 to 1:15 g:mL reported an initial increase in protein extraction at alkaline pH. The highest extraction yields of 82.6 % were achieved at solid:solvent ratio of 1:11.5 g:mL, pH 9.6, 13.5 min and 33.7 % US amplitude, and beyond these dilution the protein extraction yields reached a plateau [71].

#### 3.8.4 Particle size of matrix and other factors for extraction

Small particle sizes contribute to increase in the surface area of the solid matrix, enhancing the contact between the solvent and the solid, lessening the average diffusion path within the solid matrix of the raw material and facilitating the mass transfer during the process of extraction [100]. Particle size of pulverized dried roots of *Eurycoma longifolia* was adjusted to 0.022, 0.071, 0.223, 0.375 and 0.424 mm and a reverse relationship between the particle size and protein extraction yields was reported when using UAE [100].

Few other factors, such as dissolved gases and external pressure, also play a role in the US induced cavitation phenomenon, with the presence of gases in cavitation bubbles and optimum pressure being necessary to reach the cavitation threshold [24]. It is to be borne in mind that the varying impact of the above discussed factors on the protein extraction yield is also influenced by the differences in the nature and source of the protein extraction matrix. Further inter-related studies need to be performed involving the source of the matrix and the above-mentioned parameters to investigate their impact on protein recovery.

## 4 Use of US in combination with non-conventional techniques in protein extraction

Apart from implementing UAE techniques alone as previously mentioned, few other innovative techniques have been amalgamated with US and their combined impact on protein isolation are currently being studied, such as the use of US together with vacuum, microwaves, enzymes, membrane filtration and others.

### 4.1 US combined with vacuum

Görgüç, Özer [101] applied US together with vacuum (VU) and vacuum with enzyme (VUE) for the extraction of protein from sesame bran. The VU system operated at US (550 W) together with a vacuum system (vacuum time (1–30 min), restoration time after vacuum application (1–90 min), vacuum pressure (100–650 mmHg)) at 54 °C; while VUE system operated with US and vacuum as previously specified together with alcalase enzyme at concentrations ranging from 0.12 to 2.40 AU (Anson Unit)/100 g operating at 43 °C. The optimisation of both systems achieved high yields of protein. VU at 21 min vacuum time, 72 min restoration time and 539 mmHg was able to extract 58.0 ± 3.04 % protein; while VUE at 1.82 AU/100 g enzyme concentration, 8 min vacuum time, 68 min restoration time and 238 mmHg vacuum pressure achieved protein yields of 65.9 ± 3.04 %. Hence, it is evident that US along with vacuum and enzyme was superior to only vacuum and US application for yielding higher protein contents from sesame bran. This could be attributed to the fact that vacuum may cause the capillaries present in the cellular tissues to undergo deformation and release the gases present inside these capillaries. Therefore, when US induced cavitation occurs the tissues are disrupted and loosened allowing easier penetration of the extraction solvent that now fills up the emptied capillaries. This also subsequently allows enhanced interaction of the enzyme with the target substrates. Alcalase being a proteinase helped in disrupting protein’s interaction with other components, such as starches, facilitating its release from the bran matrix and resulting in higher yields of extraction under vacuum and US conditions applied [101].

### 4.2 US combined with microwave (MW)

Cheng, Shu [95] explored the use of US combined with MW for the extraction of protein from *Moringa oleifera* leaves. The authors used tris-HCl buffer (pH 9.0) as the solvent of extraction at 91:1 solvent: solid ratio in an ultrasonic-microwave system (US frequency of 25 kHz, US power of 300 W, MW power of 81 W) operated at 41 °C for 148 s. The protein yields of the extraction treatment using combination of the 2 technologies was 82.07 mg/g, higher than that achieved by traditional solvent based extraction using Tris-HCl buffer solution without US and MW application, which reached 68.99 mg/g. Apart from the sono-physical and sono-chemical effects of US that help in protein release from the fragmented cell walls, MW helps in extraction through dielectric heating. The electric field component of MW causes polar molecules (e.g. water) to rotate and align in both permanent and induced dipoles in a phenomenon called dipolar polarization [102]. Kinetic energy associated with vibrational, rotational and translational movement of the dipolar molecules generate substantial thermal energy. This thermal energy improves diffusional transfer of extraction solvent into the cell matrix helping in extraction [103].

On the other hand, sequential US and MW application (US at 100 % amplitude and 15 min followed by MW at 725 W for 8 min) under alkaline conditions did not achieve significant benefits in protein extraction from peanut as reported by Ochoa-Rivas, Nava-Valdez [104]. The authors found that the dual treatment gave significantly higher protein yield compared with the use of microwaves alone (725 W for 8 min), although the protein had lower purity. Moreover, when compared with US treatments alone, the protein yield and purity achieved by both methods did not have statistical differences [104].

### 4.3 US combined with enzymes

A comparative study was carried out between the use of US, enzymes or the combined effects of both on the extraction of proteins from hulled pecans [65]. The authors optimised the combined method (UE) by exploring multiple parameters that included the temperature of the process (40–60 °C), US conditions (power (100–500 W); time (5–25 min) and pulse duration (ON 5 s/OFF 3 s) and enzymatic conditions (alkaline protease addition (5,0000 U- 25,0000 U), pH (8.5–10.5), temperature (35–55 °C) and time (2.5–4.5 h) [65]. The optimum conditions for this combined method were US (400 W (1415.43 W. cm^−2^, for 15 min and 55 °C) and the enzyme application (1 % (w/w), pH 10, 50 °C, for 3 h). Only US and only enzyme application process were carried out at the above optimised parameters of UE, individually. Increase in enzyme amount till 100,000 U improved the extraction yield, facilitated by the increased interaction between the substrate and the enzyme with increasing enzyme concentration. The protein yields decreased with further increases of enzyme above 250,000 U and 3 h treatments, attributed to the increased turbidity and the complexation of inhibitors with enzymes lowering their activity. At temperature above 50 °C and pH 10, extraction yield of pecan protein dropped as higher temperature and pH denatured the enzyme impairing its activity. The highest protein extraction rates of 25.51 % were obtained under the conditions of 1415.43 W. cm-2 (400 W), 15 min, pH 10.0, 50 °C, and 1 % (w/w) alkaline proteinase, exhibiting that US and enzyme together gave higher protein yield than US or enzyme alone [65]. This was attributed to a synergistic effect of an increased surface area of the extraction material, due to surface peeling and material fragmentation caused by the US cavitation, combined with an increased mass transfer rate of cell contents due to the strong shear forces, allowing these compounds to become more accessible to the enzyme [65]. Enzymes, such as naturally present carbohydrases, may help to breakdown the complex polysaccharides present in cell wall or matrix, which may also remain bound to the proteins in the form of glycoproteins. Disintegration of the polysaccharides by these carbohydrases can help to loosen up the matrix and the tough cell walls, allowing an increased entry of the solvent and extraction of proteins. Proteolytic enzymes, on the other hand, may help to breakdown large size protein aggregates, allowing smaller protein molecules to easily get solubilized in the extraction solvent, thereby resulting in higher yields during the extraction processes. Hildebrand, Poojary [105] also showed that the use of a protease treatment combined with a 10 min US pre-treatment (100 % US power) achieved the highest protein recovery (82.1 ± 1.1 %) from the microalgae, *Chlorella vulgaris*.

### 4.4 US combined with membrane filtration

Hadidi, Khaksar [80] used US-ultrafiltration-assisted alkaline isoelectric precipitation (UUAAIP) to extract protein from alfalfa leaves. This approach consisted of US pre-treatment (US bath at 20 kHz, 100 W power, 30–50 °C temperature) coupled with alkali solubilisation (pH 9–11), followed by ultrafiltration with ceramic membranes (10 kDa, flow rate of 8–16 L/h, extraction time of 60–120 min) to separate the soluble proteins from alfalfa leaves followed by acid precipitation (pH 3.5). The optimum conditions reported by the authors were solvent:solid material ratio of 43.3 mL/g, pH of 10.1, extraction temperature of 42.5 °C, flow rate of 9.7 L/h and extraction time of 102 min. The protein contents achieved using the UUAAIP method (91.1 g/100 g) were significantly higher compared to that of the control experiments using an alkaline isoelectric precipitation method (74.5 g/100 g) and heat-coagulation extraction using steam injection at 85 °C and centrifugal sedimentation (63.9 g/100 g) [80]. Similar positive impact of ultrasonic-assisted alkaline extraction (US at 450 W, US treatment time of 84 min, US temperature of 35 °C, solid: solvent ratio of 1: 24, pH 11.5) followed by cross flow ultrafiltration of rapeseed protein using membrane of molecular weight cut-off 8 kDa was also recorded by Dong, Guo [106]. This study showed that ultra-filtered rapeseed protein extracts had higher protein content by 8.43 % and 22.62 % over the proteins which were not ultra-filtered but precipitated at pH 5.8 and 3.6, respectively [106]. While US led cavitation helped in disruption of the cellular matrix to release the proteins, ultrafiltration helped to eliminate the impurities and concentrate the proteins improving the purity of these compounds in the final extracts.

### 4.5 US combined with other techniques

**Fig. 4:**
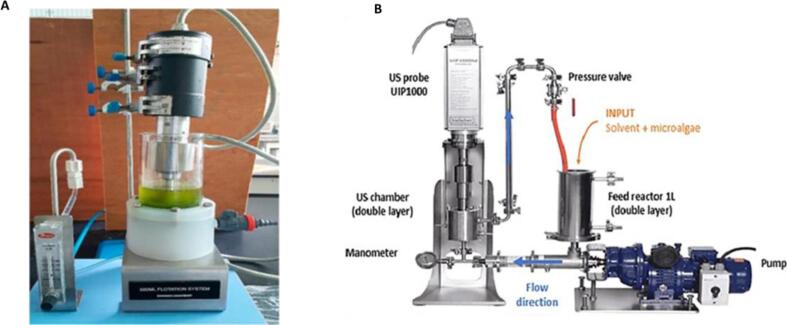
Images of a (A) display set-up of ultrasonication and liquid biphasic flotation integration process for protein extraction adapted from Sankaran, Manickam [108]; and (B) manothermosonication montage at laboratory scale in an image from Vernes, Abert-Vian [96].

US has been combined with other methods aiming to increase the yields of protein extraction from multiple sources (Fig. 4). US was coupled with the application of sugaring-out technique, a phase separation method, and supported by liquid biphasic flotation for the extraction of protein from *Chlorella vulgaris* FSP-E. Liquid biphasic flotation separates biomolecules from the feedstock by adsorbing the surface active compounds in the biomolecules on to the surface of the ascending gas bubbles, transporting them from an aqueous bottom phase to a solvent top phase [107]. This integrated approach achieved protein yields of 93.33 % at laboratory scale and 92.24 % at large scale using an optimised method operating using 0.6 % biomass concentration, 200 g/L glucose, 100 % acetonitrile, US (5 min of ON 5 s / OFF 10 s pulse mode) and flow rate 100 mL/min. The authors stated that the air bubbles generated through liquid biphasic flotation deliver an added support to the US induced cavitation as these air bubbles adsorbed surface active proteins and enabled their latter partition to the top phase, improving the separation efficiency and concentration of the extracted proteins [108].

Another promising application of US has been designed in manothermosonication which involves integrating US with pressure and temperature variation. Using this method at 2 bars of pressure, transit time of 15 s, US intensity of 55 W/cm^2^ at 24 °C, enabled to get 229 % more proteins from *Arthrospira platensis* than conventional process without US. Apart from sonoporation caused by implosion of US cavitation bubbles, tissue disintegration was intensified by the application of pressure which led to intensified fragmentation of *A. platensis* filaments and increased protein recovery [96].

## 5 Impact of US on the nutritional profile, allergenicity and degradation of alternative proteins

US is currently being used to facilitate protein recovery from alternative sources; however, the application of US can have multiple effects on the compounds extracted including modifications influencing the nutritional value of proteins (amino acid profile, protein digestibility and ANFs), their allergenicity and other negative aspects of the application of US extraction, such as degradation damage of the extracted compounds. US may influence the nutritional properties of the extracted protein determined on the basis of the amino acid composition, protein digestibility, the presence of anti-nutritional factors (ANF). Allergenicity of the US impacted proteins is also a majorly untapped yet considerably vital area of research. Table 1 highlights a summary of representative studies on the implementation of US in extraction of protein from alternative sources and its simultaneous impact on nutritional properties. Moreover, these technologies could also be used to treat and modify the nutritional properties and allergenicity associated with these alternative proteins as summarised in Table 2.

**Table 1:** Summary of representative studies on the implementation of US in extraction of protein from alternative sources and its impact on the nutritional properties of the extracted compounds.

US as pre-treatment or simultaneous application with conventional solvent based methods for protein extraction from alternative sources					
Sources	US conditions	Conventional extraction methods	Protein purity and recovery	Nutritional and physicochemical properties	References
Microalgae (*Chlorella vulgaris*)	US probe; Frequency: 50–60 Hz; Power: 100 %; Time: 10 min	Solid: solvent = 1:10 using:water (pH 7) ,HCl (0.4 M, pH 0.4) NaOH (0.4 M, pH 13.6) Control: Shaking at 170 rpm and 20 °C for 2 or 18 h using the above solvents	UAE with NaOH gave highest protein recovery (76.6 ± 0.6 %) followed by control (73.6 ± 1.2 %) using the same solvent for 18 h.	Control using water for 1 h gave highest recovery of umami free amino acids (52.32 ± 0.05 %).	Hildebrand, Poojary [105]
Macroalgae (*Ascophyllum nodosum*)	(i) US probe (13 mm probe diameter) Power: 750 W; Frequency: 20 kHz; Amplitude levels: 22.8 and 68.4 μm; Time: 10 min; (ii) US bath Time: 60 min Pellet from rehydration of seaweed powder in water was suspended in either 0.1 M HCl or 0.1 M NaOH and then treated with US	Solid: solvent = 1:15 usingacid (HCl) 0.1, 0.2, 0.3 and 0.4 Malkali (NaOH) 0.1, 0.2, 0.3 and 0.4 M Sequential 0.1 M acid-alkali and alkali-acid	Combination of first acid and then alkali extraction gave highest protein recovery (59 %); followed by single alkali extraction assisted with US at 68.4 μm (57 %).	Levels of arginine, isoleucine, leucine and tyrosine decreased through all treatment methods. Alkaline extraction resulted in higher retention of phenylalanine, serine, glycine and valine. On UAE, molecular weight of extracted protein decreased.	Kadam, Álvarez [42]
Defatted Rice bran	US probe Frequency: 20 kHz Temperature: lower than 30 °C US treatment followed by shaking at 30 °C and 200 rpm in 3 series *Series 1:* US power varied: 0, 5, 10, 15, 20 and 25 W/g of dry mass; time: 3 min. Second step time: 30 min *Series 2:* Sonication time varied: 0, 1, 2, 3, 4 and 5 min; power: 15 W/g; Second step time: 30 min *Series 3:* Sonication power and time set at 15 W/g and 2 min, respectively. Second step time varied: 0, 5, 10, 20, 30, 40, 50 and 60 min	Solid: solvent (water) = 1:10 Water extraction followed by separation of supernatant solution wherein protein was measured	Increase in power from 0 to 15 W/g, protein yield increase by 2.2 times. From 15 to 25 W/g, the protein yield slightly decreased. Highest protein yield was at US 2 min, beyond which it got slightly reduced. Protein yield gradually increased from 25.6 to 64.5 % at 20 min of second step extraction Maximum protein concentration of sample extract was 1.67 times higher than control	–	Ly, Tran [83]
Defatted soybean meal	Custom-made S-type five-frequency US Initial temperature of solution: 45 °C Frequencies: 20, 28, 35, 40, and 50 kHz Power: 120 W Sound velocity in alkaline solution: 1.5 × 103 m/s Transducer drive voltage: 220 V	Solid:solvent (water) = 1:20 pH adjusted to 11.0 followed by sonication, then pH adjusted to 4.5 for isoelectric protein precipitation	Protein extraction rate reached its maximum (73.35 g /100 g) at 28 kHz, and then it decreased with the increase in US frequency	–	Ding, Ma [53]
Soy slurry and okara	Lab-scale probe system (13 mm diameter) Frequency: 20 kHz Power output: 65 W Time duration: 0, 0.5, 1, 5 and 15 min	Milled soybean slurry in water (1:6) and okara solution in water (2.85 % solid content) individually added to a water bath at 50 ± 1 °C with 200 rpm stirring and sonicated till the desired process time was reached	On UAE, protein yield from soy slurry increased by 10 % after 1 min of treatment.	–	Preece, Hooshyar [152]
Cauliflower leaves	Optimised extraction parameters: Sonication time: 15 min Applied power: 175 W	Solvent:solid ratio = 4 mL/g, Sample sonicated after protein solubilisation at alkaline pH, isoelectric precipitation	On UAE, 12.066 g of leaf protein was obtained from 1000 g of fresh cauliflower leaves (extraction yield: 53.07 %)	Glutamic acid was the predominant amino acid. Essential amino acids represented 43.25 % of the total amino acid composition. The ratio essential:non-essential amino acids = 0.76, higher than the respective recommended values of 40 % and 0.6 of FAO/WHO	Xu, Li [153]
Bitter melon seeds	US probe Power: 300, 375 and 450 W Sonication Time: 2.50, 5.00, 7.50, 10.00, 12.50, 15.00, 17.50 and 20.0 min Pulse: ON 10/OFF 10 s Temperature: 14 ± 1 °C	Solid: solvent (1.3 M NaCl) = 1:10, sample solution sonicated after protein solubilisation at alkaline pH	From 300 to 375 W, protein extraction yield increased to 31.05 % in 17.50 min At 450 W, protein yields decreased to 28.93 % in 20 min Protein content of UAE isolates was 91.44 ± 2.57 % Extraction yield and protein content of control were 83.56 and 15.03 %, respectively.	Using UAE, aspartic acid, glutamic acid, serine, glycine, valine and isoleucine decreased	Naik, Venkatachalapathi [154]
Coconut meat	US Probe (40 mm diameter) Power: 200 W Frequency: 24 kHz Amplitude: 80 % Intensity: 6.85 W/cm^2^ Energy: 0.573 kW/kg Time: 2.5 min	Solid:solvent (water) = 1: 5 Homogenized sample treated by US, followed by precipitation at pH 4 and then separation of cream (top), skim milk (aqueous phase) and a precipitate. Cream solidified at 4 °C and defatted to get the total protein precipitate. 5 coconut meat (pulp) samples investigated	Protein yield was higher in US treated samples (49.6–86.1 %) compared to the untreated coconut pulp slurry which was the control.	–	Martínez-Padilla, Hernández-Rojas [43]
*Fusarium venenatum*	US time: 2, 4, 6, 8, and 10 min	Microfungus cultivated in Vogel’s mineral medium; separated biomass lyophilized followed by grinding (5, 10, 15, 20 and 25 min) with glass pieces and US with grinding (at same duration) to release protein.	Maximum mycoprotein release was recorded at 10 min of US with 30 min grinding (580 µg of protein content)	–	Pandurangan and Karthick Raja Namasivayam [155]
White button mushroom	US time: 10, 20 and 30 min Uniform US power Room temperature.	Mushroom powder extracted with PBS buffer (pH 7.4), followed by salting out (60 % ammonium sulphate), dialysed against PBS followed by column chromatography for purification	US for 10 min produced 228 µg/mL protein US 20 min produced 360 µg/mL 30 min produced 356 µg/mL	–	Kavitha, Damodharan [156]
Yellow mealworm larvae, field cricket adults andsilkworm pupae (defatted meal)	US probe Maximum power output: 2.5 kW Frequency: 20 kHz Amplitude: 75 % Pulse: every 3 s. Time: 20 min and aliquots were collected at 1, 2, 5, 10, 15, and 20 min.	Defatted insect coarse meal (12.5 g) mixed with 200 mL of distilled water containing 9.46 mM ascorbic acid and then sonicated.	UAE for 5 min, achieved an increased protein yield of 89 % for silkworm pupae. Mealworm and cricket had highest protein yields at 15 min showing an increase of 28 and 34 %, respectively.	No significant changes in amino acid profiles were observed on UAE. The 3 insects had similar and high levels of glutamine and minimum levels of methionine.	Choi, Wong [157]

**US applied with innovative technologies for protein extraction from alternative sources**					

**Sources**	**US conditions**	**Innovative extraction method**	**Protein purity and recovery**	**Nutritional properties**	**References**

Pecan nuts	US probe located at 1/3 of liquid volume; Frequency: 20 kHz Temperature: 40, 45, 50, 55 and 60 °C Power: 100 W, 200 W, 300 W, 400 W and 500 W with corresponding calorimetric power density: 353.85 W.cm^−2^, 707.14 W.cm^−2^, 1061.57 W.cm^−2^, 1415.43 W.cm^−2^ and 1769.29 W.cm^−2^, respectively Time: 5, 10, 15, 20 and 25 min Pulse: ON 5 s/OFF 3 s	Enzyme addition: 50,000, 100,000, 150,000, 200,000 and 250,000 U; pH: 8.5, 9.0, 9.5, 10.0 and 10.5 Enzymolysis temperature: 35, 40, 45, 50 and 55 °C Enzymolysis time: 2.5, 3, 3.5, 4 and 4.5 h	Highest protein extraction rate (25.51 %) was obtained under the optimised conditions of 1415.43 W.cm^−2^, 15 min, pH 10, 50 °C, and 1 % (w/w) alkaline proteinase.	On combining US with enzyme, the total amino acids contents increased	Wang, Wang [65]
*Moringa oleifera* leaves	US-MW collaborative extraction equipment, working with an ultrasonic frequency of 25 kHz and a fixed power of 300 W	Leaves were mixed with 0.15 mM Tris-HCl buffer (pH 9) and operated in the combined equipment followed by acid precipitation.	At optimised conditions: solvent/solid ratio: 91:1 (v/w), extraction time: 148 s, extraction temperature: 41 °C, MW power: 81 W, the yield of protein was 82.07 mg/g, higher than the control (68.99 mg/g).	LC-MS identified 19 proteins having molecular mass ranging from 10 to 90 kDa.	Cheng, Shu [95]
Sesame bran	US power: 550 W Temperature: 43 °C	Enzyme (alcalase): 0.12–2.40 AU/100 g Vacuum time: 1–30 min Restoration time after vacuum application: 1–90 min Vacuum pressure: 100–650 mmHg	Protein yield increased by 41.6 % with US, vacuum and enzyme together applied at optimum conditions compared to standard alkaline extraction Optimum conditions of VUE were 1.82 AU/100 g enzyme concentration, 8 min vacuum time, 68 min restoration time and 238 mmHg vacuum pressure	–	Görgüç, Özer [101]

**Table 2:** Summary of representative studies of the use of US to modify the nutritional properties of proteins and associated allergens isolated from alternative sources of protein.

Sources	US conditions	Effects on nutritional properties and/or allergenicity	Reference
Kiwifruit proteins	US probe Frequency: 20 kHz Power: 400 W Pulse: 50 % duty cycle Time: 0 to 16 min	Immunoglobulin E binding capacity of allergen, Act d 2 decreased by 50 % and increased IVPD	Wang, Wang [84]
Potato proteins	US assisted pH shifting treatment Protein dispersion: 20 mg/mL Triple frequencies: 20/28/40 and 20/40/60 kHz Dual frequencies: 20/28, 20/40, 20/60, 28/40, 40/60 kHz Mono frequencies: 20, 28, 40, 60 kHzIncorporation sequence of US: pre, post or online pH shifting (pHS) US time: 0, 2, 5, 10, 20, 30 min Pulse: ON 10 s/OFF 2 s Input energy density: 37.5 kJ/L Temperature: 40 °C	Using online US/T40/pHS, the digestibility rate of protein increased during gastric and intestinal digestion	Mao, Wu [113]
Rapeseed protein isolates	US Fixed frequencies: 20, 28, 35, 40, 50, 60 kHz Sweep frequencies: 20 ± 2, 28 ± 2, 35 ± 2, 40 ± 2, 50 ± 2, and 60 ± 2 kHz US times: 10, 20, 30, 40, 50 min US temperature: 25, 30, 35, 40,45 °C Power: 10, 20, 30, 40, 50 W/L Protein solutions:25 mg/mL Stirring of solution for 30 min at 25 °C, and adjusted to pH values (1.5, 2.5, 11.5, 12.5). Then, mixtures were subjected to sonication, then kept for 1 h at 25 °C), pH adjusted to 7	Reduction of lysinoalanine by 49.5 % and 74.1 %, following the use of US (28 kHz, 40 W/L, 40 °C, and 30 min) under alkali and acidic treatment, respectively	Li, Zhang [92]
Faba bean protein isolate	US probe with 8 mm diameter Frequency: 20 kHz Protein dispersions: 10 g/L	Relative protein digestibility showed a decrease of 3.6 % over control isolate	Martínez-Velasco, Lobato-Calleros [115]
Canola protein isolate	US bath Frequency: 40 kHz Power: 130 W	IVPD of US treated isolated did change significant respect to control	Flores-Jiménez, Ulloa [114]

### 5.1 Impact of US on the amino acid profile of proteins

A comparative study of protein extraction from pecan determined that the combined US and alkaline proteinase application significantly increased the total amino acid and hydrophobic amino acid contents compared to the application of proteinase alone and the control group (alkaline dissolution and acid precipitation method) [65]. The authors speculated that using this method enabled the dissociation of protein aggregates, increasing the protein recovery coupled with the enrichment of amino acid contents [65]. The influence of US on the total amino acid levels were also reported by Wang, Zhang [71] when studying pea protein. The authors reported that US-assisted alkali extracted protein (pH 9.6, 13.5 min and 33.7 % US amplitude) increased the total amino acid contents of the extracts by 12.6 % compared to a control isolate from pea [71]. Malik, Sharma [41] reported that the lysine contents of sunflower protein isolates extracted using an US probe were lower compared to those of isolates generated by US bath. The authors speculated that the probe, due to its low surface area for acoustic wave generation, provided higher US intensity compared to the US bath and that the lysine extracted was degraded under high US intensity conditions. Also, the authors reported that US induced structural changes in the protein, increasing the exposure of ɛ-amino groups of lysine, making them accessible for interaction with other functional groups of proteins and forming aggregates that will ultimately make these compounds less available to the reagents for lysine determination [41]. Zhao, Wen [87] reported that the total amino acid contents of bean (*Dolichos lablab* L.) protein generated using UAE were higher compared to those achieved using conventional alkaline extraction methods. Overall, high contents of aspartic and glutamic acids were reported in UAE extracts, suggesting that the protein may have acidic characteristics. The authors suggested that US encouraged changes in the secondary and tertiary structures of the proteins, affecting the quantity and ratio of amino acids, notably asparagine, glutamine, cysteine, and arginine [87]. Destruction of van der Waals forces and hydrogen bonds due to US cavitation and associated sono-chemical effects, are responsible for the changes in the secondary structure of proteins and the induced changes of US on the amino acid profile of the extracted compounds [87]. In the case of protein extracted using UAE from Bombay locust by Kingwascharapong, Chaijan [85], the most abundant of the amino acids were the essential amino acids - arginine, leucine, and histidine, and the non-essential amino acids- glutamic acid, aspartic acid, and alanine. Significant content of hydrophobic residues including proline, leucine, alanine, valine, phenylalanine, isoleucine, threonine and methionine, and the basic amino acids including histidine and lysine, which have been commonly linked to the anti-oxidative properties of peptides, was also reported [85]. The authors also reported an increase of umami amino acids- glutamic acid, aspartic acid, alanine, and glycine that reached 18.27 % of the total amino acid contents in protein when using UAE (20 kHz, 60 % amplitude, pulse ON 5 s/OFF 5, 20 min) [85]. However, the application of US combined with enzyme (lysozyme and protease) had little or no effect on the percentage of the umami free amino acids glutamic and aspartic acids, recovered from protein extracted from *Chlorella vulgaris* [105].

### 5.2 Impact of US on protein digestibility

Protein digestibility is an important parameter to assess the nutritional quality of a protein as it can deliver certain information about the extent of digestion and absorption of the protein and researchers analysed protein digestibility mostly through *in vitro* methods [109]. US induces physico-chemical and structural modifications in the proteins as it unfolds the compounds making them more accessible to the digestive enzymes.

Figueroa-González, Lobato-Calleros [110] explored the relative protein digestibility (RPD) for amaranth protein treated in 3 different methods - US probe (50 % amplitude, 10 min), chemical shift (pH 2 and 12) and both methods combined, using an *in-vitro* digestion system. The authors reported a significant increase of RPD in the samples treated by US alone and US coupled with pH shift 12, compared to the native untreated amaranth protein, while a significant decrease of RPD was achieved using US coupled with pH shift 2 [110]. The authors suggested that US induces protein conformational changes making them more accessible to the digestive enzymes. The lower digestibility values of the US sample combined with pH shift 2 were attributed to external factors that imbalanced denaturation-renaturation of the protein as renaturation resists digestion while denaturation facilitates digestion [110]. Lin, Liu [111] treated red lentil proteins with US alone (400 W and 20 min (ON 2 s/OFF 2 s)), enzyme (protease 1 % (w/w)) and combined US and enzyme and incorporated the treated proteins in brown rice noodles and evaluated the *in vitro* protein digestibility (IVPD) of the three noodle variants. The authors reported IVPD of 73.3, 78.6 and 80.4 % for noodles containing protein treated by US, enzyme and combined US and enzyme, respectively [111]. This indicates a cumulative effect of the conformational changes caused by US and enzymatic protein hydrolysis that led to a considerable increase in the IVPD of the products. Pan, Xu [112] also determined that the IVPD of rapeseed protein (napin) was at its highest when treated with 40 % US power. The authors also reported a decrease in disulfide bonds as the main cause of the increased IVPD together with the other protein conformational changes generated by US [112]. Similarly, Mao, Wu [113] established that online US at a mono-frequency of 40 kHz combined with pH shifting and mild heat (40 °C) could significantly increase the IVPD of potato protein by 16 % and 30.8 % during gastric and intestinal digestion, respectively, compared to the control non-US treated potato protein [113].

Conflicting results are also available on the influence of US on IVPD. For instance, Flores-Jiménez, Ulloa [114] reported no differences in the IVPD of canola protein isolates treated with US bath (40 kHz and 130 W power) compared to control samples not treated by US, that the authors attribute to the low US intensity of the US bath treatment [114]. Martínez-Velasco, Lobato-Calleros [115] reported a decrease (∼3.6 %) in the relative protein digestibility of faba bean protein isolates treated by US probe (20 kHz) over native untreated canola protein isolates. The authors considered that the decrease in protein digestibility could be associated to an increase in β- conformations as reflected by the high hydrophobic character of these protein structures [115].

### 5.3 Impact of US on ANFs

Proteins extracted from alternatives sources may contain certain ANFs or non-nutritive factors that can affect the nutritional value of the proteins [116].

Trypsin inhibitors can be commonly found in legumes, and they interact and complex with the digestive enzymes trypsin and chymotrypsin, reducing their activity, and thus, protein digestibility [117]. Vanga, Wang [118] studied the influence of US (25 kHz, 400 W, 1–16 min) treatments on soymilk proteins. The authors determined that the trypsin inhibitor activity was reduced in a time dependent manner with the US treatment, reaching reductions of 52 % over the untreated soy milk, with pulsed US treatments of 16 min, and duty cycle set at 50 % [118]. Similarly, Huang, Kwok [119] reported that the major trypsin inhibitor (Kunitz type) activity in soybeans was reduced by 5 % after 20 min of US (20 kHz). The authors attributed this effect to the disruption of disulfide bonds and other changes in the secondary structures induced by US cavitation on the trypsin inhibitors. Yang, Gao [120] also identified similar effects with US treatments inside soy tissues treating soybean seeds with US at different power levels (0 to 300 W) and germinating them in dark for 5 days. The authors reported that the trypsin inhibitor contents of soybean sprouts had a power-dependent decrease on exposure to US with maximum reduction of 98.78 % recorded at 300 W compared to the untreated control (0 W) group [120].

Phytic acid is another ANF involved in the chelation of protein and divalent mineral ions decreasing their digestibility [121], [122]. Proteins extracted from soy okara by-products using an US probe (4 kW power and 24 kJ/ L energy input, 0.5 min) had low contents of phytic acid, reaching a maximum reduction of approximately 67 % when the extraction was performed at 80 °C. In this case the authors concluded that both heat and US contributed to breakdown of the phytic acid, mainly located in the bran layer of soybean okara, by releasing the phytic acid into the solvent, reducing its content in the extracted protein precipitates [123].

Other ANFs include saponins and phenols that are responsible for the unfavourable bitter taste in the extracted proteins [124]. Polyphenols complex with proteins, reducing protein digestibility; while saponins are membranolytic against cells of the small intestine *in vivo* [125]. Moreover, saponins are also known for their iron binding properties, inhibiting the absorption of this metal [126]. When using UUAAIP (20 kHz, 100 W) for the extraction of alfalfa protein, the saponin contents of the UUAAIP extracted protein (1.56 mg/g) were significantly lower compared to those of protein extracted by only alkaline isoelectric precipitation (7.68 mg/g) [80]. The total polyphenol contents of UUAAIP proteins were 4.45 mg/g and that of the alkaline isoelectric precipitated protein was 8.58 mg/g. This implied that UUAAIP is an efficient method for elimination of saponins and polyphenols in proteins derived from alfalfa leaves [80].

The toxic secondary plant metabolite, quinolizidine alkaloid (QA) in lupin seed proteins, is known for displaying both teratogenic and anti-cholinergic effects [127]. Aguilar-Acosta, Serna-Saldivar [128] determined low levels of lupanine (30 mg /100 g protein isolate) when extracting protein from *Lupin mutabilis* using UAE (US probe, 24 kHz, 100 % amplitude (100 μm), 85 W/cm^2^ for 15 min). These levels were 58 times lower compared to those of lupin raw flour and they were also half of those measured in control protein isolates generated without US. The authors suggested that US could have liberated the alkaloids into the supernatant by disrupting the cells in the lupin seed through the violent collapse of sonication bubbles during cavitation and strong shear forces generated by this phenomenon. Thus, the QA extracted in the supernatant did not precipitate in the isolate fraction leading to the considerable decrease in its concentration in the protein isolates [128].

Lysinoalanine is an ANF composed of a cross linked amino acid formed during alkaline extraction of proteins at high pH. This ANF can reduce the availability of lysine, an essential amino acid, resulting in protein malabsorption [92]. US treatments have also shown to be beneficial for reducing this ANF. Li et al (2021) reported a reduction of lysinoalanine of 49.5 and 74.1 %, after US treatments (28 kHz, 40 W/L, 40 °C, and 30 min) performed under alkaline and acidic conditions, respectively [92].

Despite the promising results of US in inactivating these ANFs, there are still multiple biomasses currently being explored as a sources of protein and thus, multiple ANFs that would need to be explored to ensure that the UAE protein have good nutritional quality, or if they require additional US treatments post-extraction or post-isolation to ensure an efficient inactivation of these ANFs.

### 5.4 Impact of US on reduction of allergenic proteins

Proteins originating from plants, fungal spores and insects have been associated to the development of type 1 allergic reactions in humans. These allergenic protein families are mostly structural proteins, seed storage proteins, and others involved in the defence-related system or pathogenesis-related proteins [129]. Several authors have reported that US can induce powerful physical and chemical effects, altering the secondary and tertiary structures of the proteins and modifying the conformational epitopes of the allergens, enabling them to become less accessible to antibody receptors [84], [130], [131].

Few studies are available reporting the use of US alone or combined with other processing strategies to reduce the presence of allergens in alternative sources of protein. Kiwifruit is a predominant elicitor of allergy, both in adult and children due to the presence of the thaumatin-like protein, actinidin Act d 2 [84]. US processing (20 kHz, 400 W, 50 % duty cycle) for 8 min did not significantly change Act d 2 contents in the kiwi fruit proteins; however, increasing the treatment times to 12 and 16 min achieved considerable reductions of 36 % and 50 %, respectively [84]. Soy allergy is one of the most frequent allergies developed by children [132]. US treatments (20 kHz, 600 W, 50 % amplitude) for 15 min at 20 ± 1 °C reduced the allergenicity of soy protein isolates by 24.7 % [130]. Similarly, the sequential application of US (50 kHz, 2 h) followed by enzymatic treatments (chymotrypsin at 0.3 % for 4 h) was efficient to reduce the allergenic proteins Ara h 2 of roasted peanut kernels by 97.82 % [133]. Moreover, the combined use of US and MW have also been reported as beneficial to reduce allergens from castor bean (*Ricinus communis*) meal [134]. Overall, US allergen mitigation of alternative proteins is an emerging field of study and substantial studies are needed to establish a clear role of US on the specific allergen reduction mechanisms.

### 5.5 Chemical degradation of proteins extracted or treated by US

The precise mechanisms or pathways of US mediated chemical degradation of alternative proteins either during extraction or treatment have not been fully elucidated yet. However, protein oxidation (Pox) is the most common form of chemical degradation of protein that is being explored in multiple studies, mainly in relation to meat products treated or processed using US [135], [136]. Pox is induced by the reaction of free radicals with proteins in the presence of oxygen, resulting in several modifications including cleavage of peptide bonds, synthesis of carbonyl groups and protein hydroperoxides, crosslinking of proteins through formation of disulfide and dityrosine bonds and protein aggregation [137], [138], [139]. Several researchers reported that high power US led cavitation generates highly reactive free radicals and peroxides through sonolysis of water molecules which can in turn induce free radical chain reactions in food matrices that can lead to Pox [136], [140], [141]. Apart from causing modifications in the hydrophobicity and functional properties of proteins, Pox results in changes in the susceptibility of the proteins to be degraded by proteolytic enzymes, diminishing their digestibility and nutritional value [137].

Carbonyl compounds are routinely analysed as a reliable marker of Pox routinely analysed using 2,4-dinitrophenylhydrazine (DNPH) [142]. These compounds can be measured through a spectrophotometric methods, high performance liquid chromatography (HPLC) coupled to fluorescence detection and mass spectrometry, electron spin resonance spectroscopy, fluorescence spectroscopy as well as immunoblotting or enzyme linked immunosorbent assay (ELISA) [137], [143]. Protein carbonylation is an irreversible alteration attributed mainly to the oxidation of amino acid side chains constituting lysine, arginine threonine and proline [137], [144]. Moreover, certain reactive carbonyl compounds including glyoxal, methylglyoxal, 4-hydroxy-2-nonenal and malondialdehyde, formed as a result of US induced lipid oxidation, can bind to amino acids having nucleophilic groups at their active sites, such as histidine imidazole, cysteine sulfhydryl, and lysine via Michael addition and/or Schiff-base formation [143], [145], [146]. Power US has been linked as one of the factors influencing Pox in cured beef products processed under high US intensities and prolonged times [136]. Similar results have also been reported when treating porcine meat [147]. However, to our knowledge, there is a lack of similar studies analysing the influence of US treatments on the Pox of alternative proteins.

In the case of proteins rich in sulfur containing amino acids or lysine residues, the quantification of sulfhydryl and amine groups can also be efficient markers of Pox. These two functional groups can be measured using Ellman’s reagent also known as 5,5-dithio-bis-(2-nitrobenzoic acid) (DTNB) and 2,4,6-trinitrobenzene sulfonic acid (TNBS), respectively. While free or exposed sulfhydryl group, (SH_e_) can be quantified using Ellman’s reagent, total sulfhydryl group content SH_t_ can be quantified using the same method after adding mercaptoethanol. S—S or disulphide bonds formed as a result of cross linking due to protein oxidation, can be calculated numerically as (SH_t_ -SH_e_)/2 [110]. While extreme conditions of US application including high intensity or prolonged exposure were found to result in oxidation of SH groups leading to their reduction, it has also been found that at certain cases, sonication could break some of the S—S bonds in the proteins leading to the formation of new SH bonds [110], [148], [149], [150]. Figueroa-González, Lobato-Calleros [110] studied the changes in amaranth protein isolates extracted by pH shift method combined with US probe (50 % amplitude, 10 min). The authors determined lower SH_e_ contents, and higher S—S in US extracted proteins compared to that of isolates achieved using a control method. This was attributed to several factors including the oxidation of SH groups or formation of intra- or intermolecular disulfide bridges, SH/SS interchange reactions and the susceptibility of thiol groups to form mercaptide ion species (S–) under alkaline conditions [110]. On the other hand, the SH_t_ content of amaranth protein isolate increased, driven by the unfolding of the protein by US, that exposes the SH groups buried in the hydrophobic core to the surface of the molecules [110]. Similarly, a decrease of SH groups was observed in faba bean protein isolates treated by pulsed US (1200 W power, 30 % amplitude), combined with alkaline treatment [148]. The decrease in free SH groups substantiated the fact that at alkaline pH there is an increased tendency for the generation of S—S bonds when compared to the application of US under neutral pH [148]. A decline in free SH groups has also been reported for defatted wheat germ protein extracted using high US time of 70 min or more [149] and walnut proteins treated at high US power (≥600 W and 15 min) [150]. An increase in SH groups of proteins treated with US compared to untreated or control samples have been reported from multiple alternative sources of protein including soy proteins [151], brewer’s spent grain [45], pecan protein [65], rapeseed and napin proteins [112].

Despite these studies, there is currently a huge scope for explicitly investigating the possible chemical degradation processes induced by US on alternative proteins, such as Pox, through the identification and quantification of apposite markers using specific, sensitive and reproducible techniques, as well as opportunities to design processes that can curb these undesirable alterations to the proteins’ backbone and their nutritional quality.

To summarize, the mechanism involved in the US modification of the nutritive markers of protein quality encompassing amino acid profile, protein digestibility and ANF content in alternative proteins can be attributed to several factors. These factors primarily include the mode of US application, the capacity of the US to induce structural changes in the protein molecules, and the use of complementary technologies and processes, such as proteolytic enzymes, heat and pH changes of the medium. US probes, delivering higher US intensity when compared to US baths, are overall more efficient in generating strong cavitation phenomena and associated sono- chemical and sono-physical effects. These effects may induce and facilitate changes in the structure of proteins, including secondary, tertiary and quaternary structures, enabling an enhanced exposure of buried amino acid chains with the extraction solvents, resulting in overall increased protein extraction yields. The documented improvements in the digestibility of proteins may also rely on the effects of US cavitation in opening and modifying the protein’s complex structural layers, facilitating the action of digestive proteases that have an increased access to newly exposed protein structures. Breakdown of disulfide bonds by the disruptive sono-physical effects also assist in enhancing digestibility of proteins. Sono-physical effects can influence the breakdown of ANF. Proteolytic enzymes, including endo- or *exo*-proteases, aid in breaking down high molecular weight or large sized protein aggregates. These protein aggregates may exist naturally in the raw materials or they can be formed by the use of extreme US operating conditions, such as high US frequency, power or time, during the processes of extraction and product processing, as highlighted by different studies mentioned in previous sections of this review. Protein aggregates, when disintegrated by the proteases, can allow the amplified release of amino acids into the reaction mixture leading to their increased concentration in the extracts generated. Using heat along with US can also help in ANF reduction through structural disintegration of the compounds, as well as alkaline and acidic conditions of the medium can further assist in decreasing the ANF levels in the final extracts. Another interesting conclusion from the reviewed studies is that US cavitation helps in cellular tissue breakdown, also enhancing the extraction of ANFs present in different plant tissues. Subsequently, the proteins are precipitated by bringing down the medium’s pH to the isoelectric pH specific of the target proteins, while the extracted ANFs remain in the solution. Hence, the precipitate is devoid of the ANFs and the residue remaining after extraction is reduced in its ANF content and can be reused for other purposes. On the hindsight, certain harsh US operating conditions may lead to undesirable changes in the conformation of proteins. This may include increased exposure of hydrophobic amino acid groups, protein aggregation and excessive protein denaturation, which again can be further contributed by medium parameters during protein isolation such as low pH, all of which cumulatively can result in reduced protein digestibility.

Allergens, being chemically proteins, may also undergo denaturation caused by US involving significant changes in its structure and molecular properties. On the other hand, chemical modifications in US treated proteins such as protein oxidation, changes in SH-SS interactions as discussed in this review have been until now linked to the sono-chemical effects caused by the sonolysis of water molecules or through structural modification led by US cavitation. However, further in-depth research is necessary in order to understand the magnitude of the effect of US on the chemical, molecular and structural properties of the proteins that can play a significant role in its nutritive and allergenic characteristics.

## 6 Further scope of US application in the field of alternative proteins and possible strategies to mitigate associated challenges

US has manifold commercial applications for both the extraction and the modification of the nutritional properties of alternative protein isolates. Moreover, from the studies analysed in this literature review, US can be used for developing protein enriched or fortified foods from alternative sources with reduced allergenicity. In order to integrate US equipment in commercial applications, few points need to be considered. These include designing units which can deliver high power, high intensity and low frequency US waves. The use of US in continuous treatments, using flow cells, can provide large flow rates reducing the cost per volume of the treated samples; however, these treatments may require the use of multiple systems working in series or in parallel mode.

Incorporating US at pilot or industry scale faces a few other challenges as these systems and processes are often optimised at laboratory scale and cannot be scaled up directly due to huge variations in the results. On this context, Preece, Hooshyar [39] reported that UAE of soy protein extraction at pilot scale was not suitable due to the limited energy input by the pilot-scale probe system compared to the lab-scale probe used in their study. Variations in matrices, process conditions and US system set up, do play a role in the differences in the results recorded. Thus, the optimization of the specific processes for industry application has to be thoroughly researched [26]. Further improvements in energy efficiency, by improvements of US generators and transducers, will help to reduce internal heating, reducing the requirement of installing costly cooling systems [158]. Hielscher (Germany) manufactures easy to clean US devices ranging from 50 to 400 W for laboratories and from 4 to 16 kW for pilot and industrial scale processes that can be suitable for the treatment of viscous slurries of up to 250,000 centipoise.

Moreover, the prolonged use of US can also increase the incidence of food and health safety hazards. Physical hazard associated with long time US use mainly includes metallic parts falling from eroded US equipment that can contaminate the samples treated, unless controlled by proper preventive systems, such as HACCP (Hazard Analysis & Critical Control Point). Other hazards for US operators include hearing impairment on long time continuous exposure and danger of electric shock, burns and chances of electrical fire. Other risks associated to US operation include tissue and thermal injuries on high temperature rise during direct exposure, and indirect damages to the central nervous system caused by prolonged exposure to acoustic US [24], [26]. The use of soundproof systems, ear muffs, minimum equipment maintenance and training on handling US devices is necessary when operating at these conditions to avoid the development of occupational hazards. From an environmental point of view, UAE is considered a green and environment friendly technique as its use help in reducing the amount of solvents used and times required for extraction, as well as facilitating the use of water as solvent for the extraction processes instead of harsh chemical solvents [18], [19].

From the point of view of the products generated by US, proteins can get affected on exposure to extreme or harsh US conditions, as the physical and chemical effects of US can lead to denaturation and oxidative damage of these compounds, resulting in undesirable changes in the nutritional, functional and sensory attributes of proteins. An adequate temperature control and process optimisation must be in place to prevent these unwanted alterations.

Hence, US processes can be employed for their efficiency for the generation of extracts containing proteins from alternative sources, as well as to modify the nutritional attributes of these protein extracts in a green and sustainable manner catering to the growing demand of sustainable protein production systems to supply to the global population. Moreover, when collecting and analysing data for this review, it was observed that there are several other alternative protein sources on which the impact of US on the nutritional properties of proteins and their degradation, widening the need to evaluate the application of US in the field of alternative proteins beyond that of extraction.

## Funding

This research by the UCD Ad Astra Studentship (R20909), BiOrbic SFI Bioeconomy Research Centre funded by Ireland’s European Structural and Investment Programmes, Science Foundation Ireland (16/RC/3889) and the project AMBROSIA funded by the Department of Agriculture Food and the Marine (DAFM) under the umbrella of the European Joint Programming Initiative “A Healthy Diet for a Healthy Life” (JPI-HDHL) and of the ERA-NET Cofund ERA HDHL (GA No 696295 of the EU Horizon 2020 Research and Innovation Programme).

## CRediT authorship contribution statement

**Rahel Suchintita Das:** Investigation, Visualization, Writing – original draft. **Brijesh K. Tiwari:** Conceptualization, Funding acquisition, Supervision, Writing – review & editing. **Farid Chemat:** Conceptualization, Writing – review & editing. **Marco Garcia-Vaquero:** Conceptualization, Funding acquisition, Resources, Supervision, Visualization, Writing – review & editing.

## Declaration of Competing Interest

The authors declare that they have no known competing financial interests or personal relationships that could have appeared to influence the work reported in this paper.

## Data Availability

Data will be made available on request.
